# Is Sertraline a Good Pharmacological Strategy to Control Anger? Results of a Systematic Review

**DOI:** 10.3390/bs9050057

**Published:** 2019-05-23

**Authors:** Ángel Romero-Martínez, Sonia Murciano-Martí, Luis Moya-Albiol

**Affiliations:** Psychobiology Department, University of València, Blasco Ibañez Avenue 21, 46010 Valencia, Spain; soniamurciano@hotmail.com (S.M.-M.); luis.moya@uv.es (L.M.-A.)

**Keywords:** drug, sertraline, systematic review, treatment, violence

## Abstract

**Introduction:** Extensive research has made it possible to conclude that dysfunctions in serotoninergic transmission are associated with a tendency toward violence and behavioral dysregulations in humans. In this regard, it has been suggested that selective serotonin reuptake inhibitors (SSRIs), such as sertraline, which regulate the serotonin system, might reduce proneness to violence. **Aims:** This review aims to explore changes in feelings of anger-state (e.g., irritability and hostility) and anger expression as primary outcomes after sertraline treatment. **Methods:** Based on PRISMA quality criteria for reviews, a literature search was carried out through PubMed, PsycINFO, Dialnet, Psicodoc, Web of Knowledge, and the Cochrane Library. **Results:** Initially, 605 publications were identified, removing 219 duplicate manuscripts and screening the titles and abstracts of the remaining 386 records. This process left 248 articles for full-text reading, finally including 15 entries. Thus, several empirical studies were included that employed different research designs. In this regard, we considered 3 case reports, 5 open clinical trials, and 7 randomized placebo-controlled trials. The majority of the studies were unanimous in concluding that a large percentage of patients with high irritability levels responded satisfactorily to sertraline treatment. In fact, their mood improved, and they experienced a reduction in irritability and anger expression after a few weeks of treatment (approximately two weeks). However, it was necessary to increase the sertraline dose after months of treatment to avoid exhaustion effects. Moreover, not all the patients responded to the treatment and it is particularly interesting that a small percentage of patients were refractory to treatment or even showed an increase in irritability after a few weeks of treatment. In those cases, it was necessary to discontinue the treatment or reduce the dose to the initial levels. **Discussion:** Although it is necessary to be cautious about the benefits of sertraline as a way to control anger and irritability, it is relevant to consider pharmacological strategies to reduce anger-state as coadjutant treatments to psychotherapy in order to promote lasting changes in violent populations.

## 1. Introduction

Extensive research has made it possible to conclude that dysfunctions in serotoninergic transmission are associated with a tendency toward violence and behavioral dysregulations in humans, specifically with impulsive aggression and suicide [1,2,3,4]. Even though it is generally assumed that low serotonin levels explain this tendency, the serotonin hypothesis is integrated in a broader model to explain human behavior that considers the importance of this neurotransmitter system and its interactions with other neurobiological systems (e.g., vasopressin, oxytocin, adrenaline, noradrenaline, hormonal factors, etc.), contextual factors (e.g., neighbourhood, socio-economic status, caregiving strategies, etc.), and individual abilities (e.g., social, emotional, and empathic skills, etc.) to offer a broader model of violence proneness.

Therefore, substances that help to regulate the serotoninergic system might offer an interesting chance to alleviate brain functioning and enhance behavioral control. In this regard, it has been suggested that selective serotonin reuptake inhibitors (SSRIs), which regulate the serotonin system, might reduce violence proneness [5]. A large number of studies have concluded that SSRIs tend to reduce violence in approximately 8 weeks of continuous treatment [6]. It should be noted, however, that some evidence refutes this inverse association. Specifically, SSRIs have been found to double the risk of suicide in healthy adult volunteers [7] and the risk of committing a violent crime in adolescents and young adults [8].

Although SSRIs present a relatively common action mechanism, it is well known that this family of drugs differs in several aspects, such as efficacy and tolerability [9,10], which reinforces the importance of analyzing each of these antidepressants separately in order to study their effects on violence. For example, fluoxetine is one of the most well-known SSRIs, but sertraline is generally better tolerated and its effects tend to appear sooner with fewer side-effects than fluoxetine [10]. Remarkably, it has been demonstrated that, after sertraline treatment, patients reported lower anger and aggression levels in comparison with those who received fluoxetine [11], reinforcing the need to focus our attention specifically on sertraline.

Sertraline blocks the serotonin transporter into the presynaptic terminal, thus increasing serotonin synaptic levels. Additionally, this SSRI tends to decrease serotonin turnover and para-chloramphetamine-induced depletion of serotonin stores. The increase in serotonin availability for a sustained period of time entails numerous adaptive brain changes, which, therefore, lead to enhanced serotoninergic transmission. Regarding other neurotransmission systems, after chronic treatment, it has been suggested that sertraline has a minimal effect on dopamine and noradrenaline uptake, but it has been associated with a down-regulation of central β-adrenoceptors and an increase in the levels of cyclic adenosine monophosphate. Finally, it should be noted that in comparison with other SSRIs, sertraline does not present sedative effects, due to the absence of affinity for brain muscarinic and histamine H1-receptors [12,13,14].

The aim of this systematic review was to try to answer the question of whether sertraline is a good way to prevent and/or reduce human violence. This review explored, as primary outcomes, changes in several facets of human violence, such as feelings of anger and anger expression before, during, and after sertraline treatment. Finally, taking into account the existing data so far, a series of variables were considered (e.g., sertraline dose and treatment duration, psychopathological traits, patients’ gender and age, etc.) for a correct pharmacological prescription. Furthermore, the conclusions derived from this manuscript will help the scientific community, clinicians, and patients to know the available evidence on psychopharmacological treatments for violence control and their advantages and disadvantages, in order to make evidence-based choices.

## 2. Search Strategy

Based on PRISMA quality criteria for reviews [15], a literature search was performed through PubMed, PsycINFO, Dialnet, Psicodoc, Web of Knowledge, and the Cochrane Library. The study included case reports, open clinical trials, and randomized placebo-controlled trials on the role of sertraline in violence control and/or anger/irritability levels. Special attention was paid to methodological quality in order to increase the value of this systematic review. However, we decided to include not only randomized controlled trials, but also non-controlled studies, in order to compare the results of several methodological conditions.

The search strings considered relevant for this field of research and applied to both databases were **[***Sertraline***] AND [(***violence***) OR (***aggressive***) OR (***aggression***) OR (***anger***) OR (***hostility***) OR (***irritability***) OR (***offender***)]**.

All the papers selected for final inclusion met the following criteria: (a) They were empirical studies with humans (case reports, open clinical, randomized, etc.); (b) they only examined the association of sertraline with violence, aggressive behaviors, and/or anger-state; (c) there was no concomitant psychotropic medication or psychotherapeutic treatment during sertraline treatment; (d) they did not collapse the sertraline treatment group with other SSRIs; and (e) they were written in English.

Article selection was carried out by two independent researchers. The level of interrater agreement between the two researchers was 90%. In cases of disagreement, a third member of the team helped them to reach a consensus.

## 3. Results

**Figure 1:**
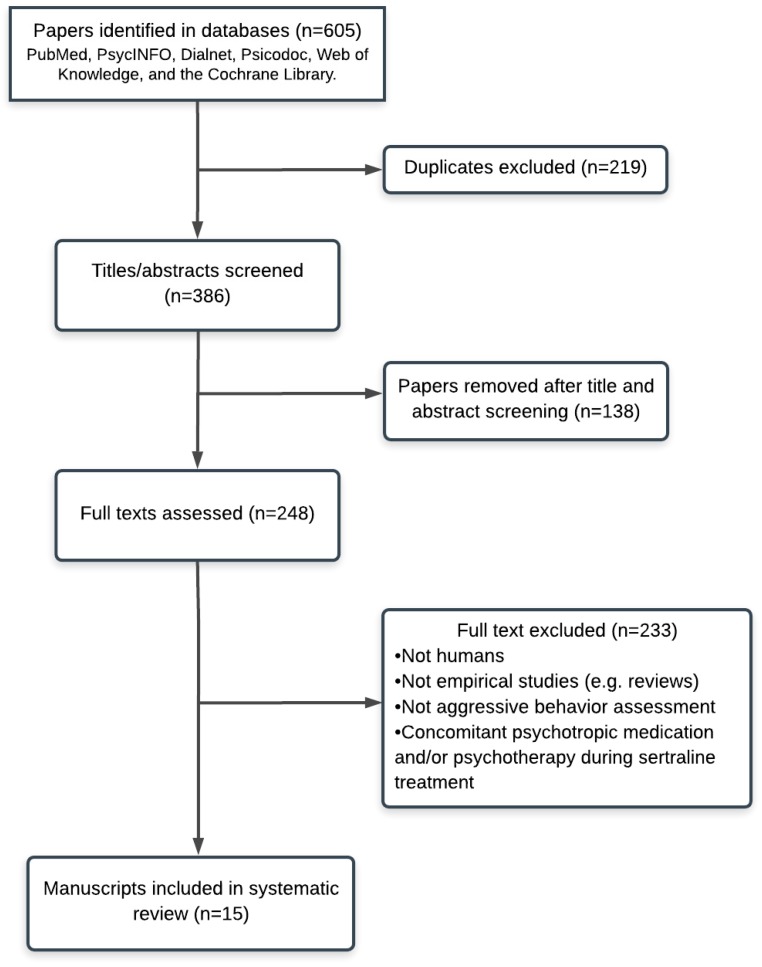
PRISMA flow chart of literature search with reasons for exclusion.

Initially, 605 publications were identified in PubMed, PsycINFO, Dialnet, Psicodoc, Web of Knowledge, and the Cochrane Library. 219 duplicate manuscripts were removed, and the titles and abstracts of the remaining 386 records were screened, leaving 248 articles for full-text reading, finally including 15 entries (Figure 1).

All the studies that were included investigated the effects of sertraline as a way to reduce and/or control anger-state, hostility, and irritability. The main characteristics of the participants and studies included in this review are summarized in Table 1 (e.g., type of design, participants’ characteristics, sertraline dosage, onset of sertraline effects, anger assessment, etc.). Initially, the main conclusions of three case reports will be presented, one describing the case of a patient with high irritability and anger expression after multiple head injuries, another describing a patient with sclerosis who presented high anger expression due to inappropriate sexual behavior, and the third explaining several cases of patients with intermittent explosive disorder. Next, five open clinical trials will be described, differentiated according to the presence or absence of depressive symptoms. These studies first describe depressive patients’ high hostility, followed by people with a high anger-state after traumatic brain injury, autistic children, participants with personality disorders, and impulsive and violent offenders. Lastly, the present study will present different randomized controlled trials with several populations, such as depressive patients, women with premenstrual dysphoric disorder, and patients with post-traumatic stress disorder (PTSD).

## 4. Case Reports

Often, these types of reports are useful for clinical practice, and they might also offer valuable guidelines for later planned experiments with a controlled design. In this review, three case reports have been included. One report describes a patient with traumatic brain injuries, and another describes a patient with amyotrophic lateral sclerosis (ALS) who presented depressive mood and behavioral alterations, including aggressive behaviors. Finally, the last study describes two men and a woman who presented intermittent explosive disorder.

The first case was a 58-year-old man who presented a history of multiple closed head injuries (vehicle accidents, seizures, and blackouts). This patient was hospitalized because he had presented depressive mood and high levels of hostility and physical aggression against others for several weeks. Although this patient presented a history of alcohol misuse for years, his alcohol-use disorder was in sustained remission. Thus, this episode of violence could not be explained by current alcohol misuse, intoxication, and/or withdrawal. Nonetheless, it should be noted that he obtained a score indicating cognitive impairment on the Mini Mental State Examination. Moreover, a brain scan revealed structural and functional abnormalities in the left occipital and adjacent left parietal areas. Based on the patient’s characteristics, specialists decided to prescribe 50 mg/day of sertraline for 7 days. After day 8, he received 100 mg/day for 2 weeks. An improvement was registered in the patient’s depressed mood and irritability after 10 days of sertraline treatment [16].

Another study portrayed the case of a 53-year-old man with ALS with frontotemporal dementia and inappropriate sexual behavior. He was depressed and presented several episodes of physical aggression toward his wife if she refused to have sexual intercourse. Professionals decided to prescribe 50 mg of sertraline twice a day. Although a marked improvement was found in the patient’s behavioral control and mood, the authors did not report how long sertraline treatment was sustained or when these improvements appeared [17].

Regarding the third case, this manuscript describes the case of three young adults whose ages ranged from 29 to 51; all of them presented intermittent explosive disorder. It should be noted, furthermore, that none of them presented a previous or current history of drug misuse or psychopathology. The two older patients (a man and a woman) started with a 50 mg daily dose of sertraline. They experienced a progressive and gradual reduction in outbursts and, in turn, an improvement in their quality of life. These effects remained for 2 years. Finally, the younger patient (29 years old) started with a minimum dose that increased to 100 mg daily for 6 weeks, with the lowest levels of anger and outburst registered during this week. These effects remained for 5 months, but after suspending the treatment, the anger increased, then decreasing progressively after 3 weeks of treatment. This man seemed to present a sertraline discontinuation syndrome that disappeared after continuing sertraline treatment [18].

## 5. Open-Label, Uncontrolled Clinical Trials

In studies of this kind, researchers and participants know what the treatment consists of, and so it is necessary to be cautious when interpreting the results. Moreover, the majority of these studies do not have a control group (e.g., placebo), which increases biases in the interpretation of the results. Based on the inclusion criteria, 5 open trials have been included, with less than 50 participants per study. Specifically, the authors analyzed whether sertraline might alleviate and/or reduce hostility in participants with various conditions, such as depression, traumatic brain injuries, and personality disorders, as well as violent offenders and children with autistic disorders.

Given that a large percentage of depressed patients tend to present dysfunctional forms of anger, Farnam et al. [19] studied whether depressed patients with higher levels of hostility would experience a decrease in several facets of anger after sertraline treatment. After 8 consecutive weeks of sertraline treatment, the authors registered a decrease in anger-state, anger feelings, and the tendency/desire to express verbal anger, control of external anger, and control of internal anger. Nevertheless, after sertraline treatment, participants did not experience changes in anger trait, aggressive behavior, aggressive reaction, presence of external anger, or presence of internal anger. Additionally, patients experienced an increase in expressing anger physically after this SSRI treatment. Sertraline was initiated at 50 mg/day and increased to a maximum of 100 mg/day, depending on patient tolerance and side-effects. Unfortunately, Farnam et al. [18] did not describe the exact moment when the changes in hostility and anger levels appeared. In fact, they only assessed the anger facets before and after the treatment. Finally, and most importantly, the authors concluded that only a minimum percentage of the severity of depressive symptoms (14%) explained the changes in the anger scales of those patients. Hence, changes in anger facets are not exclusively explained by depressive symptoms. Indeed, the following studies describe changes in anger, irritability, and hostility in patients who did not present depressive symptoms.

In fact, in addition to being a good choice for depressive treatment, SSRIs have been employed for several conditions and behavioral disturbances. In this regard, a group of men and women who experienced problems with irritability and aggression following closed head injury were voluntarily submitted to an 8-week sertraline treatment in order to reduce their violence proneness. They initially received 50 mg/day, increasing their dose by an additional 50 mg every 2 weeks, with a maximum of 200 mg/day, depending on patient response. The authors claim that participants presented a decrease in total aggression and irritability between baseline and follow-ups (after 4 and 8 weeks of treatment), but some patients experienced a slight rise in their total aggression and irritability levels between the second and final follow-ups. Nonetheless, the authors did not report whether these differences between the follow-ups were statistically significant [20].

Another study was conducted with patients of both genders with personality disorders (avoidant, antisocial, borderline, histrionic, narcissistic, and schizoid). Patients received 50 mg/day, and their dose was increased every 2 weeks with 50 mg of additional sertraline until reaching 200 mg/day, depending on patient response and side-effects. In fact, only two patients received 200 mg/day, whereas the others took 150 mg/day. Although this study was initiated with 11 patients, only 9 of them completed 4 weeks, and 7 completed 8 weeks of treatment. The patients who completed 4 weeks of treatment experienced a considerable reduction in overt aggression during the second week of treatment, with their levels being considerably lower during the fourth and eighth weeks of treatment. Regarding improvements in irritability levels, none were registered until the fourth week of sertraline treatment, and from this week to the end (eighth week), patients described a reduction in self-reported irritability. Finally, it should be noted that three patients experienced an increase in their overt aggression between the second and fourth weeks, but their levels decreased again in the eighth week [21].

One study included 34 men who were convicted of violent offenses and also presented high levels of impulsivity (assessed by a self-report). Initially, 34 men presented an adequate profile to participate in the study, but 14 left the study for several reasons (e.g., side-effects of sertraline, moving away) after 4 weeks of treatment (first follow-up), and only 20 completed the treatment schedule (12 weeks). In this study, participants received 25 mg on the first day, 50 mg the second day, and 100 mg the third day. This schedule was maintained for 3 months. The results revealed that patients presented a considerable reduction in impulsivity, anger, aggression, and assaultive behavior during the first assessment (4 weeks), and all of them were reduced at the second assessment (12 weeks). At that time, the sertraline treatment was discontinued, but researchers offered to continue the treatment under their medical supervision, which was accepted by all of them [22].

Finally, sertraline was also employed as a way to solve the irritability, anxiety, aggressive outbursts, and difficulties in tolerating transitions in autistic children. A study presented eight cases of autistic children (boys and girls) who were treated with sertraline for a long period of time to improve mood and behavioral control. However, sertraline treatment was discontinued in one boy (25 mg/day) because he experienced a strong increase in his irritability after 2 weeks of treatment. Thus, we focused on the treatment response of seven patients. The majority of the cases (75%) experienced an improvement in anxiety and less irritability and fewer aggressive outbursts after 2 or 3 weeks of treatment with 25 mg/day of sertraline. The rest of the children (25%) needed more time on the treatment for a noteworthy improvement to appear in their mood and irritability (8 weeks). Nonetheless, after the initial response to the sertraline dose, professionals decided to increase the dose to 50 or 100 mg/day because the initial effects disappeared after 6 months of treatment. However, 25% of the participants experienced an enhancement of their irritability and anger expression with the new dose, and so it was necessary to return to the initial amount of sertraline. The rest of the participants presented a good response to the dose increase, maintaining this response while the treatment lasted (approximately 12 months) [23].

## 6. Randomized Controlled Trials

Regarding depressive patients, two studies were conducted to assess whether sertraline affects anger attacks. In the first study, the effects of sertraline (50 to 200 mg/day) were compared to imipramine (50 to 300 mg/day) and a placebo in patients with major depressive disorder or dysthymia (both genders; n = 53). Results revealed that 53% of the patients who presented high baseline hostility and anger attacks and received sertraline experienced a reduction in their anger attacks. Conversely, 7.7% of the depressive patients who initially presented low levels of hostility and anger attacks and received sertraline experienced a rise in their anger attacks. This percentage was similar to the imipramine and placebo groups. Lastly, the greater the anger attacks after sertraline treatment, the lower the patient’s depressive mood improvement, although the percentage of variance explained did not appear explicitly in the paper [24].

Regarding the second study, authors conducted research with patients with major depression after mild closed traumatic brain injury. These patients received a dose that ranged from 25 to 150 mg/day (final dose average: 75 ± 39.0 mg/day). Results revealed that patients presented an improvement in their depressed mood and decreased levels of self-reported anger and aggression after 8 weeks of treatment with sertraline [25].

Another study with participants of both genders with major depression after mild closed traumatic brain injury administered sertraline and a placebo. In this case, participants initially received 25–50 mg of sertraline every morning. This dose increased progressively 50 mg every week, reaching 200 mg/day for 8–10 weeks, depending on patient’s response and tolerability. Even though there were no differences between groups in the pharmacological effect in controlling the anger levels, both groups experienced decreases in anger levels. In this regard, it is important that only 29% of the sertraline group reached 200 mg daily. In fact, the majority of them (55%) received a dose inferior to 100 mg/daily. Finally, those participants who positively reacted to treatment, improving their depressive mood, also experienced reductions in anger-state levels [26].

Sertraline has also been employed as a treatment for premenstrual dysphoric disorder. In this regard, two randomized controlled trials analyzed the role of this drug in controlling irritability and anger-state in women during this period of time. These studies compared a group of women receiving sertraline to placebo groups. In both cases, no differences were found between groups in baseline anger and irritability levels. The treatment in the first study consisted of three cycles during the luteal phase: (1) 50 mg/day, (2) 50–100 mg/day (not enough response), and (3) 50–150 mg/day (not enough response) [27]. Regarding the second study, participants received 50 to 100 mg/day when they perceived premenstrual symptoms, and they had to stop taking the pills within a few days of their menstrual flow and around the time symptoms typically ended [26]. Both studies concluded that the sertraline group presented higher reductions in anger and irritability levels in comparison with placebo groups [27,28].

Finally, two researchers studied whether sertraline improved anger control in a sample of patients with PTSD. These patients received 25 mg/day of sertraline during the first week, and their dose ranged from 50 to 200 mg/day for 12 weeks, depending on patients’ tolerability and response. Patients experienced an improvement in irritability and anger levels and emotional distress after 1 week of treatment, with its effect being greater than in the placebo group [29]. Moreover, the higher the irritability and anger levels after 1 week of treatment, the lower the response to sertraline [30].

## 7. Discussion

At the time of writing, this is the only review to summarize sertraline’s effects on reducing anger, irritability, and/or hostility, as well as anger expression. Even though it includes empirical studies with different research designs (case studies, open clinical trials, and randomized controlled trials), the majority are unanimous in concluding that a large percentage of patients with high irritability responded satisfactorily to sertraline treatment, reducing their irritability and anger expression after several weeks of treatment (approximately 2 weeks). Nevertheless, it is necessary to increase the sertraline dose after months of treatment to avoid exhausting its effects. Moreover, not all the patients responded to the treatment. It is particularly interesting that a small percentage of patients showed side-effects, including an increase in irritability and agitation after a few weeks of treatment. In these cases, it is necessary to reduce the dose or discontinue the treatment.

The results presented in our review have reinforced the hypothesis that SSRIs, in this case sertraline, are a good way to control irritability, anger, and hostility in several populations of depressed (e.g., major depression, dysthymia, depression + traumatic brain injury) and non-depressed patients (e.g., ALS, autism spectrum disorders, personality disorders, impulsive and violent offenders, premenstrual dysphoric disorder, and PTSD). It makes sense to imagine that anger level improvements might be explained by depression stabilization or SSRI sedative effects, but two studies included in this review demonstrated that depression improvement only explains a small percentage of the change in anger levels [18,25]. Therefore, based on these results, it can be concluded that depression and anger improvements tend to follow relatively different patterns of recovery. In fact, they might be considered somewhat different processes, although there is a certain overlap between them that should be kept in mind. Moreover, it is necessary to conduct additional studies to analyze whether these variables are relatively independent or not. Furthermore, many of the studies included in this review did not analyze depressive patients or depressive symptoms [19,20,21,22,25,26,27,28]. Thus, other alternative explanations for these improvements in anger and/or irritability levels should be explored.

Previous research established that there is an interrelationship between anger, anxiety, and depression [29,30], with anxiety playing a mediation role between anger and depression [31]. In this regard, it should be noted that SSRIs tend to reduce anxiety during the first week of treatment [32]. Therefore, if the aforementioned interpretation is correct, it makes sense that the reduction in agitation and hostility during the first or second week of treatment [16,20,21,25,26,27,28] could be explained by the initial anxiolytic effects of sertraline.

An interesting point of these studies that has been ignored is the reversibility or not of sertraline’s effects. In fact, Steingard et al. [20] presented an exhaustion of sertraline’s effects after several months of treatment that was compensated with an increase in the dose. The positive effects remained for 1 year, but the study did not continue after that time. Thus, it is important to study how long the sertraline effects on anger control lasted. However, treatment length should probably depend on patients’ needs, as it occurs with depressive patients.

Regarding the only study that included impulsive offenders [22], it revealed positive results, reducing not only aggressive levels, but also impulsivity. In this regard, impulsivity maintains a positive relationship with anxiety, increasing the risk of behavioral disinhibition [33]. Hence, its reduction in the first weeks might positively influence impulsivity, reducing, in turn, the risk of reacting with violence. Unfortunately, this study did not assess whether this effect was maintained or not beyond the 12 weeks that the study lasted. Curiously, this study, in comparison with the rest of the studies, only administered sertraline 3 days per week. Even so, aggressive levels and impulsivity experienced a reduction. Thus, this offers a new perspective for treating the complex phenomenon of violence in specific violent populations without depressive symptoms. Unfortunately, this study did not offer additional information about the cognitive profiles (e.g., cognitive and empathic deficits, presence of alexithymia, emotion decoding deficits, hostile cognitive schemas, etc.) of these individuals, which would help to understand the strategies these men use to cope with stress by reacting with violence. Therefore, it is particularly important to conduct research under controlled circumstances, analyzing the previously mentioned variables in order to check whether the treatment is effective or not and facilitate reinsertion of a violent population.

We initially planned to summarize the main variables that should be considered when prescribing sertraline to reduce violence proneness. Unfortunately, it would be difficult to do this, due to the reduced number of rigorous and controlled studies. Nonetheless, based on the studies included in this review, it can be concluded that sertraline is suitable for controlling anger and irritability in a large percentage of children and adults characterized by a tendency to experience outbursts, agitation, and/or irritability. A minimum dose (25–50 mg/day) for children, higher for adults (100 to 150 mg/day), is especially appropriate. However, the initial dose should be increased after several months of treatment to avoid exhaustion effects. Regarding gender, evidence was not found of a gender sensitivity to sertraline’s positive or side-effects in the studies included in this review, but this would be particularly important in future studies, in order to specify an optimal or risk profile for prescribing sertraline. Lastly, it seems that the optimal dose of sertraline to prevent anger and hostility ranged from 100 to 150 mg/day, but this dose depends on patients’ characteristics and response, and it is necessary to reach 200 mg/day in specific cases. In spite of these promising results, it must be kept in mind that only a percentage of participants in the controlled studies responded satisfactorily to the treatment. Furthermore, and most importantly, our conclusions should be interpreted with caution because the majority of the studies presented important methodological limitations.

Several limitations of the studies included in this review should be highlighted such as the lack of a homogeneous population and the limited sample size of most of the studies [16,17,18,19,20,21,22,23,24,25,26]. Moreover, not all the studies reported and/or controlled the potential confounding effects of demographic variables (e.g., educational level, economic level, ethnicity, etc.) and psychopathology assessments, among others. Furthermore, only one study [21] included children, whereas the rest only analyzed adults. Regarding the statistical analyses, none of the studies applied Bonferroni corrections for multiple comparisons. Therefore, it is highly likely that some significant results were false positives, particularly those near 0.05. Finally, there was no correspondence between the different anger questionnaires employed in the different studies. For example, some of these studies employed self-reports [19,20,21,22,23,24,25,26,27,28,29,30], but others considered families’ reports [16,17,18]. This was a potential confounding variable because not all of them evaluated the same variable. Hence, it is difficult to obtain unanimous conclusions about the association between the effects of sertraline and anger.

Finally, we cannot underestimate the risk of this antidepressant leading to suicide and/or anger expression in specific cases. Although sertraline is safe [10], three open clinical studies included in the review revealed a facilitation of anger proneness in some patients that sometimes required discontinuation of the treatment. However, it should be noted that the majority of the more rigorous studies did not report side-effects related to anger. Furthermore, we consider it particularly important to highlight that there are also patients who were refractory to sertraline. Hence, we also need to conduct additional studies in order to characterize which patients are optimal for sertraline treatment. Moreover, it would be important for future research to increase the sample size in randomized controlled trials and include not only self-report assessments, but also neuropsychological, neuroimaging, and/or psychophysiological techniques to assess factors underlying anger expression.

In summary, the present review demonstrated the importance of considering sertraline as a potential tool to diminish the risk of becoming violent by reducing anger levels in a broad population. Moreover, it is important to highlight that we should not consider depression as the main problem or assume that alleviating depressive symptoms necessarily entails an improvement in anger levels. In any case, although this SSRI seems to be a relevant pharmacological strategy to reduce anger-state, we cannot ignore the study limitations and the need to consider pharmacological strategies as coadjutant treatment to psychotherapy, in order to promote lasting changes in violent populations. Future studies should compare the effects of sertraline to other SSRIs (e.g., impipramine, fluoxetine, etc.) and serotonin norepinephrine reuptake inhibitors such as venlafaxine, which not only affect the sertraline system, but also the noradrenergic system, in order to find out whether other drugs with potent effects on other neurotransmitter systems present greater benefits for anger control.

## Figures and Tables

**Table 1:** Main socio-demographic characteristics and details about the participants in each study and the assessment methods used.

**Authors**	**Sample**	**Age**	**Gender**	**Education (Years)**	**SSRI (Sertraline) Dosage Range and Time**	**Onset Sertraline Effects**	**Current Drug Misuse**	**Violent Behavior Assessment and Changes**	**Side-Effects Related Aggressive Behavior**	**Side-Effects Not Directly Related to Aggressive Behavior**	**Funding Source**
**Case Reports**											
Kim et al. [16]	Multiple closed head injuries (n = 1), no comparison group.	58	♂	–	From 50 to 100 mg/day for unknown period	10 days	No	Family report. Reduction in aggressive outbursts.	-	-	-
Anneser et al. [17]	Amyotrophic lateral sclerosis with frontotemporal dementia and inappropriate sexual behavior (n = 1), no comparison group.	53	♂	–	100 mg/day for unknown period	–	No	Wife’s report. Reduction in aggressive outbursts.	–	–	HWP fellowship from the University of Munich
Feder et al. [18]	Treatment of intermittent explosive disorder with sertraline in 3 patients, no comparison group.	51 30 29	♂ ♀ ♂		50 mg 50 mg 50–100 mg	18 months (appeared week 2) 24 months (appeared week 6) 5 months (appeared after 6 weeks	No	Own and wife’s/husband’s report (three cases).	No	No	–
**Open Clinical**											
Farnam et al. [19]	Depression (n = 23), no comparison group. Pre-post design.	33.91 ± 14.80	♂ and ♀ (unknown gender distribution)	–	From 50 to 100 mg/day for 8 weeks	8 weeks	–	State Trait Anger Expression Inventory: Reductions in anger-state and anger control-in and -out. Absence of trait anger.	Yes	–	–
Kant et al. [20]	Closed head injury (n = 13), no comparison group. Pre-post design.	37.6	77% ♂ 23% ♀	–	From 50 to 200 mg/day for 8 weeks	4 weeks	No	Anger Irritability Assault Questionnaire: Significant reduction in irritability and aggressive outbursts.	-	-	Educational grant from Pfizer, Inc.
Kavoussi et al. [21]	Personality disorders (n = 11, only 7 completed), no comparison group. Pre-post design.	From 20 to 53	64% ♂ 36% ♀	–	From 50 to 200 mg/day for 8 weeks	From 2 to 4 weeks	No	Overt Aggression Scale: Reductions in overt aggression and irritability.	Yes	–	–
Butler et al. [22]	Violent and impulsive offenders (n = 34, only 20 completed) no comparison group. Pre-post design.	36.5 ± 11.9	♂	15.3 ± 1.7 years	25 mg sertraline (day 1), then 50 mg (day 2), and 100 mg (day 3) for 3 months	4 weeks	No	Anger Irritability and Assault Questionnaire: Reductions in the following aspects related to violence: impulsivity (35%), irritability (45%), anger (63%), assault (51%), verbal-assault (40%), and indirect-assault (63%)	–	Yes (9%)	–
Steingard et al. [23]	Autism spectrum disorders (n = 9)	From 6 to 12	67% ♂ 33% ♀	–	From 25 to 100 mg/day for 1 year	From 2 to 8 weeks	No	Parents’ report	Yes (25%)	–	–
**Randomized Controlled Trials (Multicenter, Double-Blind and Placebo Group)**											
Fava et al. [24]	Patients with atypical major depression or dysthymia (n = 53) Three groups: Sertraline (n = 56) Imipramine (n = 52) Placebo (n = 60) Pre-post design.	–	–	–	Up to 200 mg/day for 12 weeks	12 weeks	–	Anger Attacks Questionnaire: 53% of participants reported a reduction in anger attacks in the sertraline group, 57% in the imipramine group, and 37% in the placebo group	Yes (7.7%)	–	Partly funded by Pfizer Pharmaceuticals
Fann et al. [25]	Major depression after mild closed traumatic brain injury (n = 15). Participants received a week of placebo treatment, without specifying the exact moment, plus 8 weeks of sertraline treatment. Pre-post design.	41.9 ± 8.5	46.7% ♂ 53.3% ♀	15.1 ± 3.4 years	First week: 25 mg/day Second week: 25–50 mg/day Third week: 25–100 mg/day From 4th to 8th week: 25–200 mg/day	8 weeks	53% of the sample presented lifetime alcohol or drug abuse	Brief Anger and Aggression Questionnaire: Decreases in averages scores in all samples after sertraline treatment.	–	–	Educational grant from Pfizer Pharmaceuticals
Fann et al. [26]	Major depression after mild closed traumatic brain injury (n = 53). Two groups: sertraline (n = 24) and placebo (n = 29). Pre-post design.	37.5 ± 12.5	74% ♂ 26% ♀	23% did not complete high school and 77% had completed it.	Medication was started at 25–50 mg every morning. This dose increased 50 mg daily to a maximum dose of 200 mg for 8–10 weeks.	12 weeks	69% presented a history of alcohol and/or drug dependence	Brief Anger and Aggression Questionnaire: Both groups (sertraline and placebo) presented decreases in anger levels, but there were no differences between groups. In fact, there were differences between treatment responders and non-responders.	Yes (7%)	Yes (33%)	The National Center for Medical Rehabilitation Research, the National Institute of Child Health and Human Development, and National Institutes of Health grant R01HD39415. Pfizer supplied sertraline and placebo
Yonkers et al. [27]	Women with premenstrual dysphoric disorder (n = 121). Two groups: sertraline and placebo group. Pre-post design.	36.8 ± 4.8	♀	–	Three cycles during luteal phase: First: 50 mg/day Second: 50–100 mg/day (not enough response) Third: 50–150 mg/day	1 week	–	Daily Record of Severity of Problems: Significant improvements and reductions in anger and irritability for sertraline in comparison with the control group.	–	–	Pfizer Ine, New York, NY.
Yonkers et al. [28]	Premenstrual dysphoric disorder (n = 125) Two groups: sertraline and placebo group. Pre-post design.	33.7	♀	58% college studies and 32% some college	From 50 to 100 mg/day when premenstrual symptoms appeared	1 week	–	Premenstrual Tension Scale: Significant improvements and reductions in anger and irritability for sertraline in comparison with the control group.	–	–	Grants R01 MH072955, 1R01 MH072645 (Dr Kornstein), R01 MH072962, from the National Institute of Mental Health. UL1 TR000457 (Clinical and Translational Science Center at Weill Cornell Medical College) from the National Center for Advancing Translational Sciences. Pfizer supplied sertraline and placebo
Davidson et al. [29]	TEPT patients (n = 191) Two groups: sertraline and placebo group. Pre-post design.	From 18 to 65	♂ and ♀ (unknown gender distribution)	–	From 25 to 200 mg/day for 12 weeks	1 week	No	Davidson Trauma Scale (frequency and severity of anger): Greater response and mood improvements after sertraline treatment	–	–	Grant from Pfizer Inc. and NIMH grant 1R01 MH 56656 01A
Davidson et al. [30]	TEPT (n = 173) Two groups: sertraline and placebo group. Pre-post design.	From 18 to 65	♂ and ♀ (unknown)	–	From 25 to 200 mg/day for 12 weeks	–	No	Davidson Trauma Scale (frequency and severity anger): Greater response and mood improvements after sertraline treatment	–	–	Partly funded by Pfizer Inc.
